# Identification of Four Novel Synonymous Substitutions in the X-Linked Genes *Neuroligin 3* and *Neuroligin 4X* in Japanese Patients with Autistic Spectrum Disorder

**DOI:** 10.1155/2012/724072

**Published:** 2012-07-16

**Authors:** Kumiko Yanagi, Tadashi Kaname, Keiko Wakui, Ohiko Hashimoto, Yoshimitsu Fukushima, Kenji Naritomi

**Affiliations:** ^1^Department of Medical Genetics, Graduate School of Medicine, University of The Ryukyus, 207 Uehara, Nishihara, Okinawa 903-0215, Japan; ^2^Department of Medical Genetics, School of Medicine, Shinshu University, 3-1-1 Asahi, Matsumoto 390-8621, Japan; ^3^Faculty of Nursing and Rehabilitation, Aino University, 4-5-4 Higashi-Ohta, Ibaraki 567-0012, Japan

## Abstract

Mutations in the X-linked genes *neuroligin 3 (NLGN3)* and *neuroligin 4X (NLGN4X)* were first implicated in the pathogenesis of X-linked autism in Swedish families. However, reports of mutations in these genes in autism spectrum disorder (ASD) patients from various ethnic backgrounds present conflicting results regarding the etiology of ASD, possibly because of genetic heterogeneity and/or differences in their ethnic background. Additional mutation screening study on another ethnic background could help to clarify the relevance of the genes to ASD. We scanned the entire coding regions of *NLGN3* and *NLGN4X* in 62 Japanese patients with ASD by polymerase chain reaction-high-resolution melting curve and direct sequencing analyses. Four synonymous substitutions, one in *NLGN3* and three in *NLGN4X*, were identified in four of the 62 patients. These substitutions were not present in 278 control X-chromosomes from unrelated Japanese individuals and were not registered in the database of Single Nucleotide Polymorphisms build 132 or in the Japanese Single Nucleotide Polymorphisms database, indicating that they were novel and specific to ASD. Though further analysis is necessary to determine the physiological and clinical importance of such substitutions, the possibility of the relevance of both synonymous and nonsynonymous substitutions with the etiology of ASD should be considered.

##  Introduction

*Neuroligin 3* (*NLGN3*) and *Neuroligin 4X* (*NLGN4X*) are members of neuroligins expressed in the postsynaptic neurons and mediate transsynaptic signaling by interacting their ligand, neurexins [1]. Mutations in the X-linked genes *NLGN3* and *NLGN4X *(GenBank accession numbers NM_181303.1 and NM_020742.2, resp.) were first reported as being involved in X-linked autistic spectrum disorder (ASD; MIM#300425 and MIM#300495) in Swedish families [2]. Some reports have also indicated that *NLGN3* and *NLGN4X* are responsible for ASD. Mutations in these genes including 5 missense mutations [2–5], two small in-del mutations, which lead to a premature stop codon in the transcript [2, 6], an exon skipping mutation [7], and a large deletion [8], have been identified in ASD patients (Table 1). In vitro experiments using *NLGN3* and *NLGN4X* proteins carrying amino acid changes that were identified in ASD patients indicated that the gene mutations could cause ASD by a loss-of-function mechanism [5, 9]. *Neuroligin 3* p.R451C knock-in mice and *neuroligin 4X*-deficient mice exhibited autism-related behaviors [10, 11]. In addition, recent mutation screening studies revealed that the p.K378R substitution in the *NLGN4X* gene was common in individuals of different ethnicities, such as Portuguese and Greek [3, 4]. A recent association study regarding rare variants in *NLGN3* and *NLGN4X* also supported the relationship between a specific *NLGN3* haplotype and the etiology of ASD in Chinese Han male population [12]. Moreover, two synonymous variants are specifically found in ASD patients [13, 14]. 

In contrast, some data indicate that these two genes do not have a significant effect on ASD development. The frequency of mutation in the coding region was not high (<2%) among ASD patients [2–6]. It has been reported that mutations in these genes were not observed in ASD patients in the United Kingdom, Canada, the Autism Genetic Resource Exchange (AGRE) and the International Molecular Genetic Study of Autism Consortium (IMGSAC) [15–17].

Such contradictory results could possibly be due to genetic heterogeneity, regional characteristics, or differences in the ethnic background of ASD patients. It is, therefore, important to investigate the *NLGN3 *and *NLGN4X* sequences in ASD patients from various ethnic backgrounds.

Molecular screening of *NLGN3* and *NLGN4X* in Japanese patients with ASD has not yet been reported. We, therefore, performed a mutation screening study of these genes in 62 unrelated Japanese patients with ASD and 278 control X-chromosomes using polymerase-chain-reaction (PCR) high-resolution melting (HRM) and direct sequencing analyses.

##  Materials and Methods

### 2.1. Patients

The Japanese ASD patients analyzed in this study were described previously [18]. All the patients were diagnosed with ASD according to the Diagnostic and Statistical Manual of Mental Disorders (Fourth Edition criteria), the Autism Diagnostic Interview-Revised, and the Childhood Autism Rating Scale by at least two trained psychiatrists. No chromosomal aberration was found in all patients. The study was approved by the ethics committee of the University of the Ryukyus, and written informed consent was obtained from all subjects.

### 2.2. Source of Genomic DNA

Genomic DNA in patients (51 males, 11 females) and control Japanese individuals (30 males, 30 females) was isolated from successfully transformed B cells by Epstein-Barr virus using a standard protocol involving proteinase K digestion. In addition, control genomic DNA was isolated from blood of 198 healthy Japanese individuals (118 males, 80 females) using a QIA amp column (Qiagen, Hiden, Germany). 

### 2.3. Mutation Screening

Initially, mutation screening was performed in the 62 ASD subjects by PCR-HRM analysis. HRM analysis is used to scan gene variations in a PCR amplicon, for example, mutations and single-nucleotide polymorphisms (SNPs), prior to sequencing by detecting differences in the thermal denaturation of double-stranded DNA [19, 20]. We set up optimal conditions for PCR-HRM analysis of the entire coding regions of *NLGN3* and *NLGN4X*. Primers for all exons were carefully designed in flanking introns of the genes taking into account the high sequence homology between neuroligins (Table 2). Exons 2, 7, and 8 of *NLGN3* and exons 2, 5, and 6 of *NLGN4X* were amplified in two or three segments to obtain a fine resolution of the HRM curves. Only an upstream part of exon 2 (exon 2.1 in Table 2) of *NLGN4X* was sequenced in all ASD patients because we failed to distinguish between the complicated SNPs located in the region.

### 2.4. Condition of PCR-HRM Analysis

PCR was performed under optimized conditions using Ex-Taq DNA polymerase (TAKARA Bio Inc., Japan) as follows: 20 *μ*L reaction mixture containing Mg^2+^-free 1 × PCR buffer (TAKARA Bio Inc., Japan), 0.25 mM dNTPs (TAKARA Bio Inc., Japan), 0.25 units Ex-Taq DNA polymerase, 1 *μ*M of each primer, and the optimal concentration of MgCl_2_ was loaded per well in a 96-well PCR plate (Roche, Basel, Switzerland). The amount of genomic DNA used in each reaction was 40 ng for males and 20 ng for females. Fluorescent DNA dye, SYTO 9 (0.5 *μ*M; Life Technologies, Carlsbad, CA), was added before amplification. Genotype analysis of each sample was performed in duplicate. The melting curves were sequentially analyzed using LightCycler 480 Gene Scanning Software (Roche, Basel, Switzerland) to detect sequence variants. Evaluation of the HRM curves was sequentially confirmed by direct sequencing (Life Technologies, Carlsbad, CA).

### 2.5. Screening in Controls

Variants identified in ASD patients were then investigated for detecting the common variants by searching the database of Single Nucleotide Polymorphisms (dbSNP) build 132 and the Japanese Single Nucleotide Polymorphism database (JSNP). All variants found in ASD patients were scanned in genomic DNAs isolated from EBV transformed cells or blood cells in control Japanese individuals. 

### 2.6. Assessment of the Substitutions

The effect of the substitutions was evaluated using Mutation Taster software (http://www.mutationtaster.org/).

##  Results

 We identified four variants, a synonymous substitution and three intronic substitutions, in *NLGN3 *in the 62 ASD patients. The synonymous substitution (c.1698G>A, p.K566K) was observed in a male ASD patient. We also identified four variants, comprising three synonymous substitutions and a substitution in the 5′ UTR region of *NLGN4X*. One synonymous substitution, c.297C>T (p.G99G), was observed in a female patient, and two substitutions, c.516C>T (p.I172I) and c.1590C>T (p.F530F), were observed in two different male patients. The variants identified in this study are summarized in Table 3. Nonsynonymous substitutions, including seven substitutions reported previously, were not identified in the Japanese patients by PCR-HRM analysis (Tables 1 and 3). The four synonymous and an intronic substitution were not found in the control X-chromosomes from unrelated healthy Japanese individuals, without any neuropsychiatric disorder (Table 3).

##  Discussion

By using PCR-HRM analysis we identified an intronic and four exonic substitutions only in 62 Japanese patients with ASD, two out of 62 patients (3.2%) in *NLGN3* and three out of 62 patients (4.8%) in *NLGN4X*. The exonic substitutions comprised one synonymous substitution in *NLGN3* and three synonymous substitutions in *NLGN4X* (Table 3). The PCR-HRM analysis could detect 90% of the sequence variations with 100% accuracy [19, 20]; therefore; we were able to identify almost all the changes in *NLGN3 *and *NLGN4X* in these patients. 

In this study, we analyzed genomic DNAs from EBV-transformed cells in patients with ASD. The source of genomic DNA, especially extracted from EBV-transformed cells, should be considerable in each experiment, because there is a possibility that unexpected substitutions occur during the transformation [21, 22]. Analyses in control genomic DNA showed that there was no sequence alteration or substitution bias in the exons of *NLGN3* and *NLGN4X* between genomic DNA isolated from blood and EBV-transformed cells in this study (Table 3). 

Our mutation screening study in Japanese ASD patients failed to detect novel nonsynonymous mutations and seven known nonsynonymous mutations that were identified in previous studies [2–5] as well as a study in a Chinese Han population [12]. Considering the low frequency of nonsynonymous substitutions in these genes seen in previous reports [15–17], a larger number of ASD patients should be sampled. Nevertheless, our results suggest that nonsynonymous substitutions in *NLGN3* and *NLGN4X *may account for only a small proportion of Japanese patients with ASD. In addition to ASD, there are some reports indicating that *NLGN3 *and *NLGN4X* are also relevant to Asperger syndrome [2], X-linked mental retardation [6], and Tourette syndrome [8]. Considering the function of *NLGN3 *and *NLGN4X* [1], additional mutation screening studies in such neurobehavioral disorders should be needed in near future.

 Experimental evidence is increasing that synonymous substitutions could affect the protein function through transcription or translation impairment [23–25]. While the clinical and physiological importance of the four synonymous substitutions and an intronic substitution are not clear at this moment, Mutation Taster software (http://www.mutationtaster.org/) [26] evaluated that the substitutions might affect protein structure by altering splice site. According to previous reports of mutation screening in *NLGN3* and *NLGN4X*, two synonymous mutations, p.Y74Y in *NLGN3* and p.A558A in *NLGN4X *(Table 1), were also observed in ASD patients but not in healthy controls [13, 14]. A recent study in a Chinese Han population has indicated that a common intronic variation in *NLGN3* may influence the susceptibility of males to ASD [12]. Although further analysis is necessary to demonstrate the biological effects of synonymous and intronic substitutions on ASD, these substitutions as well as nonsynonymous substitutions should be taken into account. 

##  Conclusion

We identified four substitutions, one in *NLGN3* and three in *NLGN4X*, specific to Japanese patients with ASD. They were synonymous but the possibility of the association of both synonymous and nonsynonymous substitutions with the etiology of ASD should be considered.

## Figures and Tables

**Table 1:** Previously identified sequence variations in coding regions in ASD patients.

Gene	NT^1^ change	A-A^2^ change	Mutation type	Ethnic background	Ref.^3^	NCBI
*NLGN3*	c.222C>T	p.Y74Y	Synonymous	Finnish	[13]	NM_181303.1
	c.1351C>T	p.R451C	Missense	Swedish	[2]	NM_018977.3

*NLGN4X*	c.259C>T	p.R87W	Missense	Irish and Scottish	[5]	NM_020742.2
	c.759G>A	p.G99S	Missense	Portuguese	[3]	NM_020742.2
	c.1186 insT	p.D396X	Frameshift	Swedish	[2]	NM_020742.2
	c.1253delAG	p.D429X	Frameshift	French	[6]	NM_020742.2
	c.1597A>G	p.K378R	Missense	Portuguese and Greek	[3, 4]	NM_020742.2
	Not described^4^	p.A558A	Synonymous	German	[14]	NM_020742.2
	c.2574C>T	p.R704C	Missense	Portuguese	[3]	NM_020742.2
	del exon 4	In-frame^5^	Skipping	AGRE^6^	[7]	NM_020742.2
	del exon 4–6	Truncated^7^	Large deletion	Irish and English	[8]	NM_020742.2

^1^NT: nucleotide.

^2^A-A: amino acid.

^3^Ref.: reference number.

^4^The number of substituted nucleotides was not mentioned in the reference.

^5^Exon 4 skipping mutation was predicted to result in an in-frame exclusion of 62 amino acids.

^6^Autism genetics resource exchange.

^7^The translated protein was predicted to be entirely truncated between exon 3 and exon 6.

**Table 2:** Primer sets used to screen for variants by PCR-HRM analysis.

Gene	Exon	Forward	Reverse
*NLGN3*	2.1	GCTCAGTTTTGAGGTTCAAGTC	TCACTGGGCAGTGGTACTCG
	2.2	CACAGTCAACACTCACTTTGG	GATGGTTAGAAGCATTTTCACAG
	3	GGCAGAGGCCTCCTGTTATT	CAAATCCTCCCTGCAAGGCA
	4	TGGCTTGCTGGGCCACACTG	GCCAAAGACAGATGAACAGCC
	5	AGGTTGAGCAACCCCATGAGT	GGGCCAGAGGATAACACCATT
	6	CATCCCTCTGCCTTCATTGTC	TAGAAGAGAGCTGGCCGATTC
	7.1	CAGCCTCAGTGACAAAGGAAT	CAGGGTGTCCTTACCCTCAG
	7.2	GTAAGGACACCCTGCGAGAG	TGGGGTCTCAAAGAGGAAAA
	8.1	GTGACCCCAGATTTCCATGT	GGCCAGAACGTTAAGGAACA
	8.2	ATCACCCGCAGGCCCAATGG	CCTCACACTCGTGGTGGGTG
	8.3	GGAGGAGCTGGCAGCATTAC	CTGGAGATTGGCTGTGCTCT

*NLGN4X*	2.1	AAAGCCCTATCTCTCTGCAGG	TGAGTAGTATTTCGGATGCCAG
	2.2	AAGAACACCGTTACCCAATGAG	GAGACATTATAAAACCCTCCTAG
	3	TTAGCATTGGTGAGTCAGTGTG	CCGTCAAAACGAGAAGTGGACT
	4	CTTTTTCTATTTGGCCACCA	TTCTTGGTTCAGGGTATTTGC
	5.1	AGCTGCATTTCTGTCCTGTG	TCTCCCGCAAAGTGTCTTTC
	5.2	CCAACTTCGTGGACAACCTT	ACCCCAACACGAAGATGAAC
	6.1	CACGTCACATGTGGAAGAGT	GACGGCAATGGTGACACTTA
	6.2	TCCTCATTGAAACCAAACGA	AACATTCCTGGTCTGGAGAC

**Table 3:** Sequence variants identified in Japanese patients with autistic spectrum disorder.

Gene	Exon	SNP ID^1^	Location	Translation	ASD^2^		Control^3^	
					Male	Female	EBV cell line	Blood
*NLGN3*	4	—	c.567+22C>T	Intronic	0/51	1/11	0/90	3/278
		—	c.567+52C>T	Intronic	10/51	2/11	12/90	32/278
	5	—	c.727+47G>C	Intronic	1/51	0/11	0/90	0/278
	7	—	c.1698G>A	p.K566K	1/51	0/11	0/90	0/278

*NLGN4X*		rs6639602	c.-305-86T>G	5^′^UTR	0/51	1/11	1/90	4/278
	2	—	c.297C>T	p.G99G	0/51	1/11	0/90	0/278
	3	—	c.516C>T	p.I172I	1/51	0/11	0/90	0/278
	5	—	c.1590C>T	p.F530F	1/51	0/11	0/90	0/278

^1^Reference number of the variant documented in dbSNP build 132 or JSNP. (—) indicates that the variant does not have a reference number.

^2^Number of ASD patients (50 males, 11 females) with the variant.

^3^Number of control chromosomes with the variant in EBV transformed cell line (30 males, 30 females) and blood (118 males, 80 females).
